# 4th ISCB Latin American Student Council Symposium: a virtual and inclusive experience during COVID-19 times

**DOI:** 10.12688/f1000research.28330.1

**Published:** 2020-12-15

**Authors:** Camila Castillo-Vilcahuaman, Catalina Valdivia, Cleidy Osorio-Mogollón, Claudia Silva-Andrade, Rafael Puche, Sebastián Ayala-Ruano, Yesid Cuesta-Astroz, R. Gonzalo Parra

**Affiliations:** 1 Facultad de Ciencias y Filosofía - Universidad Peruana Cayetano Heredia, Lima, Peru; 2 Institute of Environmental Sciences, Faculty of Biology, Jagiellonian University, Krakow, Poland; 3 Universidade de São Paulo - Faculdade de Medicina de Ribeirão Preto, Sao Paulo, Brazil; 4 Laboratorio de Biología de Redes, Centro de Genómica y Bioinformática, Facultad de Ciencias, Universidad Mayor, Santiago, Chile; 5 Unidad de Estudios Genéticos y Forenses, Instituto Venezolano de Investigaciones Científicas (IVIC), Caracas, Venezuela; 6 Universidad San Francisco de Quito, Colegio de Ciencias Biológicas y Ambientales (COCIBA‐USFQ), Quito, Ecuador; 7 Colombian Institute of Tropical Medicine - CES University, Medellin, Colombia; 8 European Molecular Biology Laboratory, Heidelberg, Germany

**Keywords:** student council, iscb, conference, virtual

## Abstract

Since 2014, the ISCB Latin American Student Council Symposium (LA-SCS) serves as the main biannual activity where students from all levels, postdocs and early researchers from the entire Latin American region can gather to discuss recent advances in the fields of bioinformatics and computational biology. This time we faced a major unexpected obstacle, a worldwide pandemic that has completely disrupted human activities at a planetary scale. Countless conferences have been either canceled, reprogrammed for the next year or moved to a virtual format. However, thanks to an important strengthening of the Latin American student network and the creation of several new RSGs in the continent, we were able to get together a fearless team that aimed to overcome the pandemic obstacles and still organise the 4th LA-SCS. Here we summarize our experiences in our first virtual symposium.

## Introduction

The Student Council (SC) is the student branch of the ISCB (International Society for Computational Biology). As a global organization, it aims to nurture and connect the future generations of computational biologists ( Parisi *et al.*, 2019; Shome *et al.*, 2016; Shome *et al.*, 2019). To reach different regions around the globe, the SC created the Regional Student Group (RSG) program, that aims to create satellite organizations around the world. Latin America joined this initiative in 2012 and Latin American RSG groups have experienced a considerable expansion since then.

In 2014, the first edition of the Latin American Student Council Symposium (LA-SCS) was held in Belo Horizonte, Brazil ( Parra *et al.*, 2014). LA-SCS is a great opportunity for Latin American students to connect and engage in bioinformatics research, creating an important network in countries in which the field of bioinformatics is still new. Since the first edition, LA-SCS has been steadily held every two years ( Monzon *et al.*, 2017) fuelling the appearance of country specific symposia as well that are important for the local communities of the Latin American RSGs ( Geraldo *et al.*, 2018; Parra *et al.*, 2016).

In 2014, the ISCB-SC published an editorial article, showing how some young RSGs had to learn from their failures and mistakes to overcome adversity while giving their first steps in organizing scientific events ( Mishra *et al.*, 2014). Leaders from RSG Argentina described how they faced several incidents and obstacles, in 2012, when organising their first-ever student meeting. Many years later, a global phenomenon has posed a major problem for most scientific events in the world. 2020 has been the year of a pandemic that closed any opportunities to travel or to engage in congresses around the world. This forced several congresses and symposia to go virtual, a format not well explored until this year. However, in contrast to the Argentine experience in 2012, after several years of networking and many RSGs that had been created in the region, a large group of highly experienced leaders as well as new ones that have joined the organisation recently, are in an unprecedented shape to face this huge challenge. In fact, the organizing team of the LA-SCS saw this as an opportunity to engage with a major portion of Latin American students who normally would not be able to afford travel expenses.

A big advantage this year is that never before has there been so many Latin American RSG groups. There are currently nine Latin American RSGs (Argentina, Brazil, Chile, Colombia, Costa Rica, Ecuador, Mexico, Peru, and Venezuela) and all of them have actively participated in the organization and promotion of this event. LA-SCS 2020 went virtual with all the resources and expectations to be a unique encounter between Latin American students and researchers, using virtual resources as a way to reach a bigger audience.

## Scope and format of the symposium

The format of the LA-SCS has been a day-long meeting, usually preceding the main ISCB-LA conference. However, with the COVID-19 pandemic, the meeting was held virtually, using the AirMeet platform for conferences and poster presentations. In total, we had 10 oral presentations and 15 poster presentations. We were also delighted to have three renowned researchers as keynote speakers: Janet Kelso (Max Planck Institute for Evolutionary Anthropology), Cath Brooksbank (European Bioinformatics Institute - EMBL-EBI) and Alejandro Sanchez-Flores (Universidad Nacional Autónoma de México). We took advantage of this new virtual platform to invite researchers that may inspire the Latin American student community.

The symposium had 50 participants. We received 27 submissions from students that were peer-reviewed by 6 independent reviewers. 10 abstracts were selected for oral presentations and 15 additional abstracts were accepted for poster presentations. Poster presentations may be found on the website of ISCB-LA SoIBio BioNetMX Symposium 2020 ( Table 2).

In addition, we offered five fellowships for speakers and attendees who demonstrated financial need. Two presenters and three attendees were awarded with fellowships that covered the expenses for LA-SCS2020 registration. All beneficiaries were Latin American students and the organising team took the decision based on motivational letters and curriculum vitae from all interested individuals.

## Student and early-career researchers’ presentations

### Oral presentations

The event hosted 10 oral presentations, all from Latin American students (see Table 1 for full details about presenters and titles). From these, four were PhD students, two were MSc students, and four were undergraduate students. SARS-COV-2 was also present. Four of the 10 presentations were related to the virus. For oral presentations, the distribution of topics was more uniform, counting with five works related to structural bioinformatics and five related to genomics. Presenters were asked to record a 15 minute video of their presentation, which was transmitted during the symposium, and they had 5 minutes to answer questions from attendees.

**Table 1.** Oral presentation titles and topic.

Title	Topic	Presenter
Local Frustration Improves Protein-Ligand Docking Predictions.	Structural bioinformatics	Camila M. Clemente
Edible vaccine design against SARS-COV-2.	Structural bioinformatics	Juan Manuel Martínez Villalobos
Molecular docking of SARS-CoV-2 RNA polymerase reveals potent inhibitors of replication machinery than the current repositioned drugs.	Structural bioinformatics	Cleidy Osorio Mogollón
Flavonoids as potential inhibitors of SARS-CoV-2 Main Protease.	Structural bioinformatics	Gabriel Jiménez-Avalos
Understanding viral linear motifs tethered by disordered linkers.	Structural bioinformatics	Juliana Glavina
An insight into the Rhizosphere Microbiome in constructed wetlands.	Metagenomics	Alejandro, Llanos-Lizcano
Mutation frequency in breast cancer cases from 3 cities of Colombia.	Genomics	Alejandro Mejia Garcia
System analysis of Leishmania ncRNAs through developmental stages.	Genomics	J. Eduardo Martinez
Homophily and Heterogeneity in the Cancerous Cell Line Network.	Genomics	Diana García-Cortés
Updated HLA frequencies provided SARS-CoV-2 epitopes for South America.	Genomics	Aldhair Médico

**Table 2.** Poster presentation titles and topic.

Title	Topic	Presenter
Engineering Subunit Vaccine against Human Respiratory Syncytial Virus.	Structural bioinformatics	Abu Tayab Moin, Moin
A multi-epitope vaccine against the highly pathogenic SARS-CoV-2.	Structural bioinformatics	Jose Marchan
Engineering Novel Epitope Based Vaccine against Epstein-Barr Virus.	Structural bioinformatics	Abu Tayab Moin, Moin
Computational study on curcumin analogs as anti-inflammatory drugs.	Structural bioinformatics	Nusrat Afrin Anamika
Epitope-based multivalent,multipathogenic vaccines against DENV & ZIKV.	Structural bioinformatics	Hiya Islam
Improving Geomfinder algorithm: profilings, benchmarks & performances.	Structural bioinformatics	Alejandro Valdés-Jiménez
Immunoinformatics Approach to Epitope-Based SARS-CoV-2 Vaccine.	Structural bioinformatics	Subyeta Sarwar
FrustratometeR: An R package to calculate energetic local frustration.	Structural bioinformatics	Atilio Osvaldo Rausch
BGCViz: a shiny app for different BGC annotations' visualization.	Genomics	Pavlo Hrab
Comparative genomics of Clostridia isolated from gut microbiota.	Genomics	Claudia Silva
A bioinformatic approach to improve wet-lab PCR.	Genomics	Javier, Anleu Alegría
Chromatin states from simplified epigenetic data.	Genomics	Leandro Murgas
Posters link: https://www.iscb.org/cms_addon/conferences/lascs2020/viewinghall.php?track=Structural Bioinformatics&day =poster#search		

### Poster presentations

The symposium featured 12 poster presentations (see Table 2 for full details about presenters and titles). Six were undergraduate students, three were MSc students, and three were PhD students. It is important to highlight the fact that despite the event being hosted by a Latin American country and promoted as a “Latin American Symposium”, half of the participants of the poster session were from other continents. Of these intercontinental participants, four were from Bangladesh (one of them presented two posters), and one from Ukraine. This was possible due to the event being hosted on-line, which reduced the traveling and registration costs associated with the participation, closing an important breach associated with sharing knowledge and networking. The event was held on-line because of the current SARS-COV-2 pandemic, and this topic was not absent from the poster presentation session. We had two posters related to the development of vaccines for SARS-COV-2. Regarding the topics of the poster presentations, eight of them were based on structural bioinformatics, while four of them were based on genomics. The participants were asked to send their poster as a pdf file, and a three minute recording of themselves explaining their work, which were then made available on the ISCB YouTube channel in the playlist “ Latin American Student Council Symposium 2020”. There was also time allotted for presenters to answer questions and interact with attendees during the symposium.

## Keynote speakers

The first keynote speaker was Dr. Cath Brooksbank who is head of the EMBL-EBI Training Programme. Her talk was entitled "The accidental bioinformatician". In this, she talked about her scientific journey, from Bachelor degree studies until her current position as Head of Training at EMBL-EBI, and her works with CABANA Project ( https://www.cabana.online/), ISCB Education Committee and other institutions. She highlighted the importance of the training, as a heart EMBL-EBI mission, mentioning the different institutions in Latin America that make this work possible. In addition to this, Dr. Brooksbank explained about the slow implementation of data-driven biology by creating a sustainable capacity-building programme, and the challenges in relation to this. Finally, she talked about indicators of quality and impact of her programme training.

The second keynote speaker was Dr. Janet Kelso who is head of the Bioinformatics research group at the Max-Planck Institute for Evolutionary Anthropology in Leipzig, Germany. Her talk was entitled "Using Ancient DNA to understand modern human history". Dr. Kelso presented several historical highlights in the understanding of modern human evolution. She told us about the timeline through which fossils were found from modern humans as well as from Neanderthals and Denisovans and how techniques were improved to be able to recover genetic material from them. Dr. Kelso described introgression events between Neanderthals and *Homo sapiens* and also between Denisovans and Neanderthals showing that modern humans crossed with each other in the past. She finished her talk by giving a summary of Neanderthal variants in present humans and how they can lead to deleterious traits or positive ones.

The third keynote speaker was Dr. Alejandro Sanchez-Flores who is head of the Sequencing and Bioinformatics Unit in Universidad Nacional Autónoma de México (UNAM). His talk was entitled "Microbiomes, probiotics and health: Comparative metagenomics applications". The aim of his talk was to give attendants an insight on his research, focusing on how metagenomics can aid in the characterization of probiotics. Dr. Sanchez-Flores presented his work with the cotija cheese, a Mexican cheese, that presented a unique microbiota. His work can help cotija cheese producers in the future. At the end of his talk, he also gave attendants a brief tour about what metagenomics is and its methodology.

## Award winners

The LA-SCS Symposium awarded three prizes: one for the best oral presentation and two for the best poster presentations. Winners for the Best Poster/Oral Presentation Awards sponsored by ISCB were selected by the organizing committee members who filled up an online form with their preferences.

The oral presentation prize went to Aldhair Médico from Universidad Peruana Cayetano Heredia who was selected for his talk titled “Updated HLA frequencies provided SARS-CoV-2 epitopes for South America” ( Requena *et al.*, 2020). This work rectifies and presents the current scenario of allelic distribution of Human Leukocyte Antigens (HLA) in South American populations by millions of data points. With that information, we used the top immunoinformatics software to predict region-specific T linear epitopes in the SARS-CoV-2 proteins. These results address the regional genetic misrepresentation and can help combat the current pandemic of COVID-19.

The poster presentation prizes were received by Alejandro Valdés-Jiménez from Universidad de Talca, Chile who presented “Improving Geomfinder Algorithm: profilings, benchmarks & performances”. This presentation was about the improvements made to the Geomfinder algorithm. The methodologies (patterns for parallel programming) and several techniques that were applied (profiles, benchmarks, among others) were shown, as well as the results (accelerations) obtained. And Leandro Murgas from Universidad Mayor, Chile for his work “Chromatin states from simplified epigenetic data''. This work is summarized as follows: Structural changes of chromatin modulate access to DNA for all proteins involved in transcription, a process linked to variations in epigenetic marks. Importantly, chromatin can be classified in different functional states depending on the pattern of these marks. The objective was to reduce the number of epigenetic marks that are required to assign chromatin states by using machine learning to identify relationships between different marks. Initial results indicate that it is possible to predict epigenetic marks based on other marks, thus, reducing the experimental data required to assign chromatin states.

## Conclusions

This was the first virtual LA-SCS thanks to unusual conditions such as the COVID-19 pandemic. Overall, it showed a great benefit for Latin American students, who could present their work without worrying about costly travels to another country. Interestingly enough, some students presented research based on their work done while working from home, which suggests how bioinformatics provides ample flexibility to researchers to work from any place.

A virtual format of course has some disadvantages. When people attend in person meetings, they dedicate a full set of days to participate in that event. In a virtual format, people usually divide their attention between the Symposium and other work/family related activities and hence don’t attend the full program. Also, there is a huge impact in the networking possibilities. If this format is maintained in near editions, we need to find ways to engage more participation and networking among the attendees.

However, we found that online conferences held several advantages. Geographical distances disappear as a barrier to a lot of people that would not be able to afford the associated travel expenses, which is a major problem in a continent as large as Latin America. In the same direction, we were able to feature worldwide recognised researchers as keynotes, representing a great opportunity for Latin-American students to engage with important international research. Another positive side was that we could have recorded talks. This meant that any student that still felt unsure about their speech skills could be comfortable presenting their work at the symposium with a pre-recorded version of it. One big advantage about pre-recorded talks is that the attendees can ask questions while the video is being played and the authors can answer immediately, leaving more space for discussion at the end. Also, it is worth noting that pre-recorded talks help to maintain the time schedule without time delays.

Works related to the COVID-19 pandemic were especially abundant during this symposium. This showed the adaptability of bioinformatics studies, because most of these works were performed using information made available by other research groups and deposited in databases of public access just a few months in advance. It also showed how Latin American bioinformatics students can contribute to ongoing research to and to understanding biological mechanisms involved in COVID-19 pathogenesis.

Finally, the organization committee showed a great diversity, with members being from different countries in Latin America, showing different perspectives on the needs and interests of Latin American students in their respective countries. We do not know if virtual conferences are going to become the main way to organise scientific gatherings but it is clear that a format that has been resisted before the COVID-19 pandemic offers a lot of advantages and it was much easier to organise than initially thought. Virtual conferences take down the economic and geographical barriers that impact greatly on those students coming from small universities and institutes across our continent. Many students and young scientists have been able to attend our events for the first time because of this virtual format. We are sure that after the COVID-19 pandemic situation is over, we will keep online conferences as a new and complementary tool to keep expanding our community to all those interested students and young researchers in the region. Overall, the first virtual LA-SCS showed great flexibility and inclusivity. The flexibility offered by the online format was reflected in the works done at home as a consequence of this pandemic. The inclusivity was reflected in the attendance of students and young researchers from different parts of Latin America and the world, some of which would not have been able to take part in an on-site event.

## Data availability

No data is associated with this article.
